# A Perspective on Studies of Phage DNA Packaging Dynamics

**DOI:** 10.3390/ijms23147854

**Published:** 2022-07-16

**Authors:** Philip Serwer

**Affiliations:** Department of Biochemistry and Structural Biology, The University of Texas Health Center at San Antonio, San Antonio, TX 78229, USA; serwer@uthscsa.edu

**Keywords:** key biomedical problems, metastatic cancer, neurodegenerative diseases, phage procapsids, phage therapy, RNA and DNA phages

## Abstract

The Special Issue “DNA Packaging Dynamics of Bacteriophages” is focused on an event that is among the physically simplest known events with biological character. Thus, phage DNA (and RNA) packaging is used as a relatively accessible model for physical analysis of all biological events. A similar perspective motivated early phage-directed work, which was a major contributor to early molecular biology. However, analysis of DNA packaging encounters the limitation that phages vary in difficulty of observing various aspects of their packaging. If a difficult-to-access aspect arises while using a well-studied phage, a counterstrategy is to (1) look for and use phages that provide a better access “window” and (2) integrate multi-phage-accessed information with the help of chemistry and physics. The assumption is that all phages are characterized by the same evolution-derived themes, although with variations. Universal principles will emerge from the themes. A spin-off of using this strategy is the isolation and characterization of the diverse phages needed for biomedicine. Below, I give examples in the areas of infectious disease, cancer, and neurodegenerative disease.

## 1. General Features of Studies of Nucleic Acid Packaging via a Procapsid

The Special Issue “DNA Packaging Dynamics of Bacteriophages” focuses on how bacteriophages (abbreviated, phages) package genomic nucleic acid when the packaging occurs by pre-forming a phage capsid (often called a procapsid) and, then, drawing the nucleic acid molecule(s) into an internal cavity of the capsid. Packaging of this type occurs in viruses with both double-stranded RNA and double-stranded DNA genomes. An attraction for studying this process is that it is complex enough to be biological (includes energy transduction via ATP cleavage), but simple enough so that one can envisage using current technology to obtain a thorough answer to the question of how it occurs (reviews [1,2,3,4,5,6,7,8]). As I discuss below, an additional attraction for me is that I can imagine that, from studies of phage nucleic acid packaging, solutions to key problems of biomedicine will evolve. Given the importance of solving these problems for those who suffer from various diseases, translational focus on these diseases is appropriate.

The concept of procapsid-dependent phage DNA packaging first landed on the platform of rigorous studies when a group at Caltech showed [9] that, in vivo, a radiolabeled phage T4 capsid packaged alternatively radiolabeled DNA that had been synthesized after assembly of the capsid. Density labeling and buoyant density centrifugation were then used to find that the capsid did not disassemble and then re-assemble during packaging [9]. A group at the University of Toronto followed with an in vitro experiment in which infective phage T7 particles were produced when a T7 procapsid was added to and incubated with a capsid-deficient extract of phage T7-infected cells [10]. The number of infective particles produced was linearly related to the concentration of the procapsids. This showed, again, that the capsid drew a genome to its interior and did not disassemble and then re-assemble during packaging [10]. These experiments attracted others into pursuit of the details of DNA packaging. One of the first details learned was that the capsid of the mature phage was physically and chemically different from its procapsid precursor (reviews [1,2,3,4,5,6,7,8]).

Given the rigor and simplicity of the initial experiments, one might imagine that the details were learned quickly and without major difficulty. In fact, this product of the imagination is not accurate. The key, progress-limiting problem was obtaining and characterizing capsid-DNA packaging complexes in all intermediate states of packaging. The manuscripts in this symposium describe some of the most recent work to determine the details. Several packaging systems are described in these manuscripts. The systems vary in the aspect of packaging dynamics accessed. One might say that they vary in the window through which packaging dynamics is observed. Integration of the information obtained is a long-range goal.

In doing this, Schrödinger’s essay, “What is Life”, is worth considering in that it describes a general relation of biology to the physical sciences. The basic theme of this essay is that physical systems, of the size of biological systems, inevitably lose order and detectably and spontaneously decay toward randomness. This process is quantified via the concept of entropy, the increase of which quantifies the increase of disorder. However, in contrast, biological systems maintain order to a much higher degree [11]. One of the (physical science-oriented) founders of phage biology, Max Delbrück [12], has speculated that the complexity of theories to explain biological retention of order is obscuring a more fundamental principle [13]. An informative analogy is the complexity of 19th century theories to explain atoms in terms of Newtonian mechanics, with regard to specific heats, for example. The more fundamental principle being obscured by these theories was the description of atom-associated electrons by standing waves that had quantized wavelengths [14]. That having been said, at least one other biology/biochemistry-proficient physicist has expressed the opinion that complexity is the entire story for biology, i.e., no new, post-quantum mechanics principle will be found beyond genetic selection [15,16]. A goal for the future is a general and fundamental explanation (independent of biochemical details) of how order is maintained in biology.

## 2. Phages and Phage DNA Packaging in Relation to Biomedicine: A Perspective

### 2.1. Phage Therapy of Infectious Disease

The most obvious use of phages in biomedicine is in the phage therapy of bacterial disease. That topic has been discussed extensively (recent reviews [17,18,19,20,21,22,23,24]). Here, I make the point that the following biomedicine-wide lesson is, in my opinion, worth learning from the low productivity [17,18,19,20,21,22,23,24] of recent efforts in phage therapy. As noted for DNA packaging above, strategy should be focused on identifying and solving the key problem that inhibits progress. We have made the points that (1) this problem is phage lifetime (also called phage persistence) in blood that is low and (2) persistence is highly variable among different phages, even phages that are related [25]. In other words, the lower the persistence is, the lower the chance that a phage will have a therapeutic effect. Conversely, high persistence (2–6 h) for a lytic phage should result in effective phage therapy until bacteria become either sequestered (in a biofilm, for example) or resistant to the phage being used. Bacteremias should all be susceptible to phage therapy done with a high persistence, lytic phage. I do not understand why persistence has not been a focus of both researchers and funding agencies.

Obtaining high-persistence, lytic phages can be achieved through at least four procedures: (1) screening of newly isolated lytic phages, a process that overlaps with new phage isolation and screening for investigations of DNA and RNA packaging, (2) selection for high-persistence mutants of a low-persistence phage, (3) chemically or physically converting low-persistence phages to high-persistence phages, and (4) genetic engineering-based conversion of low-persistence phages to high-persistence phages. Procedure (2) has been successful in the case of phage lambda in murine blood [26]. Procedure (1) has the best prospect for requiring minimal time and resources but requires high-throughput isolation of new phages. A key objective is to develop procedures for the rapid and inexpensive screening of phages with a high probability of having high persistence. If physical phage characteristics are used for screening, simultaneous screening for windows-on-DNA packaging can be performed, thus tightly linking basic science with biomedical objectives.

### 2.2. Metastatic Cancer

Might a process of “key problem ignoring” also be confounding modern research on curing metastatic cancer? Although this situation is more complex, the answer appears to be yes. For example, a major component of current work on chemotherapy has the goal of finding compounds that specifically inhibit cancer cell biochemistry while doing minimal damage to healthy cell biochemistry [27,28,29,30,31,32]. The target is often DNA repair or DNA replication because of the more rapid DNA replication of tumor cells [28,29,30,31,32]. However, so far, toxicity plagues all chemotherapeutic drugs, and metastatic cancer cells eventually evolve drug resistance. Metastatic cancer is reported to be the cause of 80–90% of cancer-derived deaths (reviews [33,34]). Toxicity and drug resistance evolution are the key problems to solve.

Toxicity and drug resistance have biased recent anti-cancer efforts toward immunotherapy and viro-, immunotherapy. These are also problem-burdened strategies, although with some successes (reviews [30,35]). Most therapeutic problems of virotherapy fall in the category of low persistence [35]. Finally, an alternative approach to the limitations of past chemotherapy is to generate anti-tumor activity via bacteria that (1) selectively colonize tumors and (2) deliver immunostimulation and bacterially synthesized anti-tumor compounds. Selective colonization occurs, at least in part, because of immune-suppressed and hypoxic tumor environments. However, this approach also has problems of toxicity (reviews [36,37,38]). Its use also appears to have the disadvantage of conflicting with antibiotic usage in the control of infections.

In order to improve chemotherapy (which is the most often used anti-tumor therapy [33,34]), one might continue down the path of isolating additional drugs that are cancer cell-selective inhibitors. This strategy runs the obvious risk that past limitations are an indication of a natural conspiracy that is not to be bypassed. At some point, a natural conspiracy becomes a natural law. Or, using Schrödinger’s focus on antinomies, one might propose that past chemotherapy is crippled by the following antinomy. Cancer cells arise via evolved biochemistry. We try to cure cancer by doing things that place pressure on these cells to evolve. Basically, the key problem has not been solved.

A chemotherapeutic, anti-cancer, key problem-oriented strategy is to deliver currently available anti-cancer drugs so selectively that the tumor-delivered amount can be increased enough to destroy the tumor before drug resistance has a chance to evolve. However, the current circumstance in this area is reminiscent of the current circumstance in the area of phage therapy of infectious disease. Focus is not on the most logical solution to key problems. A result is that key problem-oriented chemotherapeutic strategies, such as this one, are typically in the background if considered at all [27,28,29,30,31,32,33,34].

One possible way to implement this latter strategy is to achieve *high* drug-delivery selectivity via compounding of several, *independent low* selectivities. In theory, this can be achieved by using a drug delivery vehicle (DDV; reviews [39,40,41]) that is gated. Gating a DDV introduces a major advantage for achieving tumor-selective drug delivery. (1) Open the gate to load the drug in the DDV. (2) Close the gate to tumor-deliver the drug–DDV without leakage of drug into blood (i.e., dramatic decrease in toxicity). (3) Rely on the relatively high porosity of tumor-specific blood vessels and inefficient lymphatic system of tumors (EPR effect [41,42,43]) to achieve tumor-entry selectivity (first low-selectivity event). (4) Either open the gate again or disrupt the DDV selectively in tumors (second-level low-selectivity event) and (5) use a drug that has low selectivity for operating in the conditions of a tumor (third low-selectivity event; examples in [44,45]). Liposomes are currently the most often used DDV [39,40,41] but are not gated and leak drug when in blood [46].

However, from studies of phage nucleic acid packaging, we know that all double-stranded DNA and RNA phages have procapsids and mature capsids with a nucleic acid-entry portal [1,2,3,4,5,6,7,8], potentially useful as a DDV-gate. This portal is discussed in the articles presented here. Phage T4 has been shown to be gated for ethidium [47]; a phage T3 capsid has been shown to be gated for GelStar [48]. Both these phages exhibit high murine persistence [25], which is potentially as critical for a DDV as it is for phage therapy of infectious disease. Each newly isolated phage has the potential for having improved characteristics, including an increased EPR effect, for use as a gated DDV. To bypass problems with adaptive immunity to phage-DDVs used for more than a week [49,50], phage-DDVs from several unrelated phages will be needed. Again, high-throughput phage isolation and screening is supportive, as anticipated for studies of DNA packaging.

The importance of specificity compounding is emphasized by the finding that, in humans, the EPR effect is often less selective than it was once thought to be. In humans, drug efficacy increase via the EPR effect is typically high enough to detect only for Kaposi sarcoma and head and neck cancers (review [41]). The EPR effect is stronger in murine xenografts [41]. Nonetheless, DDV encapsulation has produced wider detectable benefits via reduction of toxicity to humans, cardiotoxicity in the case of Doxil (reviews [41,42]).

### 2.3. Neurodegenerative Disease

Together with multi-drug-resistant bacterial disease and metastatic cancer, modern medicine has been confounded by neurodegenerative diseases: for example, Alzheimer’s, Parkinson’s, ALS, Huntington’s, and prion-caused diseases (reviews: [51,52,53]). Most current neurodegenerative disease models, therapy and prevention are not effective [52,53,54]. The key problem to solve is not even apparent from most of the current literature.

**Figure 1:**
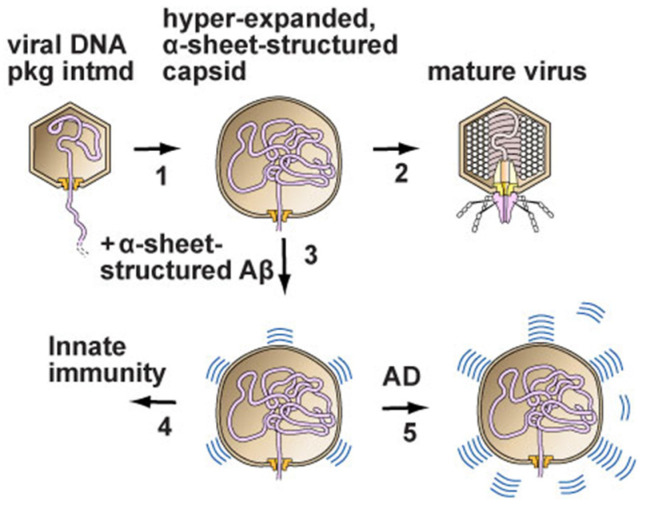
A skeletal model of phage T3 DNA packaging and a derivative model for the virus initiation of neurodegenerative diseases. (1) Packaging of phage T3 DNA involves hyper-expansion of the capsid’s outer shell when packaging via a packaging ATPase (not included in the drawing) slows. The hyper-expansion occurs via the taking by outer shell subunits of α-sheet conformation. α-sheet is a protein conformation that can be visualized in two dimensions by rotating every other amino acid in a β-sheet by 180°. (2) Packaging is completed, with the capsid contracting and, finally, a tail (host-adsorption organelle) being placed on the capsid. (3) In the case of virus (herpes virus, for example) infection of a human, the α-sheet-containing intermediate stimulates an innate immune response in which amyloid protein assumes α-sheet structure and extends the α-sheet of the packaging intermediate, thereby inhibiting packaging and inhibiting the assembly of progeny viruses. (4) Innate immunity is the result of (3) being achieved as programmed. (5) Excessive production of α-sheet-amyloid has a toxic effect that is the source of Alzheimer’s disease (AD) and other neurodegenerative diseases [55,56]. Recent work suggests that toxicity is exerted through damage by transfer of α-sheet-amyloid to lysosomal membranes [56]. The figure is adapted from reference [55]. A direct linkage is suggested of biomedicine to the basic science of DNA packaging.

One hypothetical model is based on disease causation via an innate immune over-reaction to protein conformation associated with virus infection. DNA packaging is, perhaps, the source of this conformation, according to one version of this hypothesis [55,56]. A possible culprit has been identified in the case of phage T3 DNA packaging [55] (illustrated in Figure 1), which suggests a direct overlap of basic science with biomedicine. The possibility of causation by anti-microbial innate immunity obviously needs further work but is generally not being pursued despite evidence that previous virus infections are associated with the subsequent appearance of Alzheimer’s disease [57,58]. Again, each newly isolated phage has the potential to assist understanding and, indeed, therapy [56].

## 3. Strategy for Solving Key Problems of Biomedicine: Ramifications of Studies of Phages

Phage-based solutions to problems of biomedicine are potentially achieved via two strategies that differ in concept but are likely to be complementary to each other if both are used in proportion to need. The first strategy is to re-engineer known phages, either genetically (via either selection or genetic engineering) or physically/chemically. This strategy, alone, will eventually work in theory.

However, in practice, the details may be difficult to achieve with known, perhaps favored, phages, although immediately accessed for other phages. The following are illustrative examples in the case of studies of DNA packaging. Phages T3, T7, and phiII are related in both genome organization and genome sequence [59]. However, their procapsids vary dramatically in stability. The phiII procapsid is so unstable [60] that procapsids, in general, are unlikely to have been discovered if phiII had been the only phage studied. The phiII procapsid completely converts in less than a day to a phage-like capsid during/after ultracentrifugal purification and even while in lysates of infected cells [60]. Similarly, capsids with incompletely packaged DNA are easily isolated for phage T3, but not for phage T7 [61].

The second, complementary strategy has been discussed above. (1) Efficiently (in time and cost) isolate phages as diverse and as numerous as possible. (2) Efficiently screen each phage for utility in biomedicine and basic science. A key problem to solve is developing screening procedures that are efficient and accurate enough to be practical. (3) Then, use the phages with the most appropriate characteristics. The above observations with T3, T7, and phiII suggest that this strategy will be useful for “opening windows” on the various events of phage nucleic acid packaging.

The following are advantages of using this second, new phage-based strategy. (1) The use of natural evolution, rather than either genetic engineering or laboratory evolution, unlinks the search from dependence on what is known and improves the probing of the unknown. (2) The screening involved can simultaneously (and efficiently) provide a foundation for both basic science and biomedicine. The screening procedures include, at the least, native gel electrophoresis and ultracentrifugation. Even if genetic or physical/chemical manipulation of known phages is used, screening still has to be done, although via a process more costly, in time and resources, than screening environmental phages. Thus, screening of environmental phages appears to be the most efficient of the above strategies.

In other words, environmental phage “fishing expeditions” have very high potential in basic science and medicine, especially with the current database technology to record and transmit data. A compulsory death sentence for “fishing-expedition phage grants”, if employed, would also (1) be a death sentence for many people and (2) suppress the probing of the unknown.
